# Personalization of the Immunosuppressive Treatment in Renal Transplant Recipients: The Great Challenge in “Omics” Medicine

**DOI:** 10.3390/ijms16024281

**Published:** 2015-02-17

**Authors:** Gianluigi Zaza, Simona Granata, Paola Tomei, Alessandra Dalla Gassa, Antonio Lupo

**Affiliations:** Renal Unit, Department of Medicine, University of Verona, Piazzale A. Stefani, 1, 37126 Verona, Italy; E-Mails: paola.tomei@univr.it (P.T.); alessandra.dallagassa@gmail.com (A.D.G.); antonio.lupo@univr.it (A.L.)

**Keywords:** renal transplantation, pharmacogenomics, pharmacogenetics, personalized medicine, immunosuppression

## Abstract

Renal transplantation represents the most favorable treatment for patients with advanced renal failure and it is followed, in most cases, by a significant enhancement in patients’ quality of life. Significant improvements in one-year renal allograft and patients’ survival rates have been achieved over the last 10 years primarily as a result of newer immunosuppressive regimens. Despite these notable achievements in the short-term outcome, long-term graft function and survival rates remain less than optimal. Death with a functioning graft and chronic allograft dysfunction result in an annual rate of 3%–5%. In this context, drug toxicity and long-term chronic adverse effects of immunosuppressive medications have a pivotal role. Unfortunately, at the moment, except for the evaluation of trough drug levels, no clinically useful tools are available to correctly manage immunosuppressive therapy. The proper use of these drugs could potentiate therapeutic effects minimizing adverse drug reactions. For this purpose, in the future, “omics” techniques could represent powerful tools that may be employed in clinical practice to routinely aid the personalization of drug treatment according to each patient’s genetic makeup. However, it is unquestionable that additional studies and technological advances are needed to standardize and simplify these methodologies.

## 1. Ensuring Long-Term Graft Survival in Renal Transplantation: A Matter of Adequate Immunosuppression

Renal transplantation is the gold standard therapy for patients affected by end stage renal disease, a clinical condition characterized by severe biological/biochemical alterations that require renal replacement therapy to ensure patients’ survival, and it is followed, in most cases, by a significant improvement of patients’ quality of life, reduction of medical expenses and prolongation of life [1,2,3]. In fact, immediately after transplantation, patients experience rapid and significant changes of their clinical conditions and undergo considerable physiological modifications [4,5].

However, sometimes, transplanted subjects may undergo important clinical complications (e.g., infections, malignancies and cardiovascular diseases) most of the time drug-induced. In fact, the actual employed immunosuppressant medications, although valuable against short-time graft complications (including acute renal rejection), are still less effective to ensure long-term graft survival. As reported, death with a functioning graft and chronic allograft dysfunction (CAD) are still elevated, with an annual rate of 3%–5% [6].

CAD is defined as a functional and morphological deterioration of a renal allograft at least 3–6 months after transplantation due to non-immunological (mainly calcineurin inhibitors-induced nephrotoxicity) and immunological (e.g., chronic antibody mediated rejection-CAMR) factors [7].

During this process, any anatomical component of the allograft kidney may undergo fibrosing/sclerosing changes: (a) glomeruli may increase progressively in the matrix and/or may develop segmental or global sclerosis; (b) arterial intima may be affected by fibrosis, and accumulation of hyaline material in small arteries and arterioles; (c) peritubular capillaries may develop multilayered basal lamina, and there may be thickening of glomerular capillary walls with reduplication of the basement membrane and mesangial interposition and (d) the downstream interstitial matrix could increase resulting in interstitial fibrosis and collagen scar accompanied by progressive tubular atrophy [8].

In addition, substantial evidence implicates major histocompatibility complex alloantibodies as a cause of late graft loss [9]. Capillary deposition of C4d, a split product of the classical pathway of complement activation, is an established marker of antibody-mediated allograft rejection early after transplantation [10,11,12,13,14]. In acute rejection, this complement split-product has been shown to be closely associated with the presence of alloantibodies. C4d was also identified in renal allograft biopsies with morphologic signs of chronic rejection [13,15].

It seems clear that, in this context, a correct administration of immunosuppressive drugs, minimizing/avoiding the onset and development of all above-mentioned graft damages, and maximizing its ability to control rejection, may result in a significant improvement of the graft and patients’ survival.

However, at the moment, the methodologies to adjust the dosage of these drugs with a narrow therapeutic index (such as calcineurin inhibitors, mammalian target of rapamycin inhibitors and antiproliferative medications) generally provide inadequate, non-reproducible and poor predictive of efficacy/toxicity before drug administration results [16].

**Figure 1:**
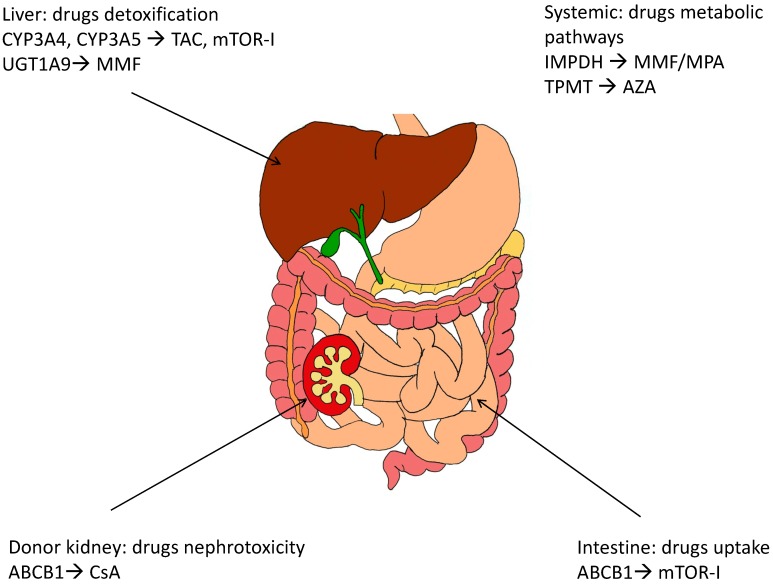
Tissue localization of major polymorphic enzymes involved in metabolism and disposition of immunosuppressive drugs. TAC: Tacrolimus; mTOR-I: mammalian target of rapamycin (mTOR) inhibitors; CsA: Cyclosporin A; MPA: Micophenolic acid; MMF: Mycophenolate mofetil; AZA: Azathioprine.

Additionally, as largely described, inherited differences in drug metabolism and disposition and genetic variability in therapeutic targets (e.g., receptors) need to be taken into account because of their role in modulating drug effects and toxicities [17,18,19] (Figure 1).

For these reasons researchers and clinicians worldwide are working together to identify minimally invasive biomarkers useful to personalize therapy based on patients’ genetic/genomic characteristics, to select valuable molecular tools for drug monitoring and to recognize biomarkers able to early identify patients at high risk to develop chronic graft damage in which an early therapeutic change may alter transplant outcomes.

It is expected that, during the coming years, the use of “omics” techniques may have a critical impact in the transplant field facilitating the achievement of these crucial objectives.

## 2. Understanding the Genetic Influence in Drug Response: A First Step to Personalize Immunosuppressive Treatment in Renal Transplantation

**Figure 2:**
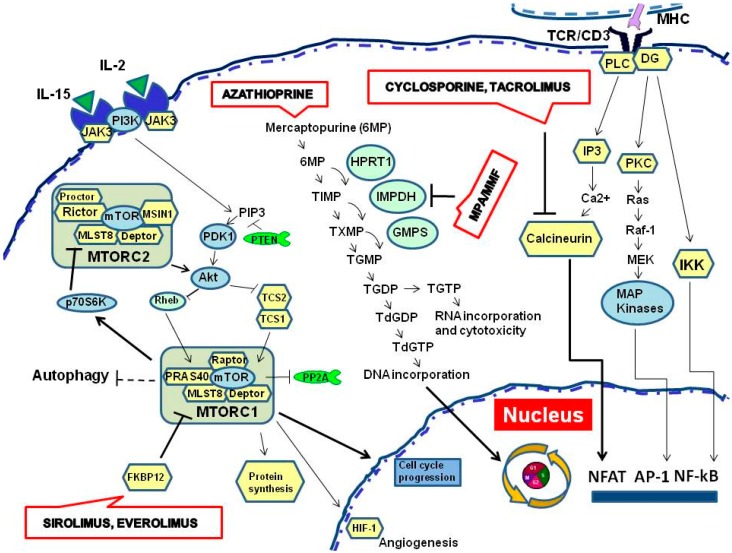
Mechanisms of action and targets of immunosuppressive drugs used in renal transplantation. MPA: Micophenolic acid; MMF: Mycophenolate mofetil.

The current therapy of renal transplantation employs several immunosuppressive agents (Figure 2 and Table 1), most of the time combined, commonly classified according to their mechanism of actionsCalcineurin inhibitors (Cyclosporine A, Tacrolimus); Inhibitors of purine synthesis (Mycophenolate mofetil/Mycophenolic Acid and Azathioprin); Mammalian target of rapamycin inhibitors (Sirolimus and Everolimus).

These drugs are frequently co-administered with glucocorticoids (Methylpredinosolone, Prednisolone) and, during induction therapy, with monoclonal or polyclonal antibodies (e.g., Basiliximab, Tymogobulin) [20].

### 2.1. Calcineurin Inhibitors (CNIs)

CNIs, cyclosporin A (CsA) and tacrolimus (TAC), currently employed as the backbone of most immunosuppressive regimens in renal transplantation, have similar mechanisms of action.

Briefly, CsA acts by complexing with cyclophilin and, after the inhibition of the activity of phosphatase calcineurin, prevents the translocation of nuclear factors of activated T cells (NFAT) into the nucleus with a subsequent inhibition of the transcription of several genes encoding for cytokines and other immune mediators, particularly IL-2. TAC acts through immunophilin FK binding protein-12 (FKBP12) to produce downstream inhibition of the same pathway. Inhibition of calcineurin by these agents blocks T-cell activation and, consequently, immune response propagation [21].

Although both drugs share a similar mechanism of action, and the same drug transporter and metabolizing enzymes are involved, important differences exist between them (Table 1).

TAC is mainly metabolized by two enzymes of the cytochrome P450 family, CYP3A5 and CYP3A4 [22,23]. Therefore disposition and dose to blood concentration ratio of TAC seem to be influenced by the polymorphic genetic variants of these two genes.

The main determinant is a single-nucleotide polymorphism (SNP) in intron 3 of CYP3A5 (6986A>G; SNP rs776746), also known as CYP3A5*3. CYP3A5*3 allele is a splice variant with a premature stop codon and encodes an enzyme with a reduced activity. Patients homozygous for this variant require a dose of TAC approximately 50% lower to reach the blood target concentration compared with carriers of the CYP3A5*1 allele (wild-type) [24,25,26,27]. This condition has a major clinical impact considering that around 5%–15% of white individuals are expected to express CYP3A5*1, whereas approximately 30% of Asians, and 70% of individuals of African descent express this variant [28]. Therefore, a correct genotyping for *CYP3A5*, while on the waiting list for transplantation, could be useful to optimize TAC dosage in the post-transplant period [29,30].

Then, the *CYP3A4* polymorphisms may also affect TAC pharmacokinetics. The CYP3A4*22 (rs35599367; c.522-191C>T in intron 6) SNP has been linked to reduced CYP3A4 mRNA expression and lower *in vitro* CYP3A4 enzyme activity [31]. In renal transplant recipients, the CYP3A4*22 T-variant allele was associated with a reduced TAC dose requirement, independent of *CYP3A5* genotype [32].

Additionally, the CYP3A4*1B SNP involves an A to G transition on promoter region of *CYP3A4* (−392A>G) and has been linked to an increased CYP3A4 activity. Tavira *et al.* [33] report that, at one year post-transplant, the patients who were CYP3A5*3/*3 + CYP3A4*1B carriers had TAC C_0_ values in the target range, whereas those carrying the CYP3A5*3/*3 + CYP3A4*1/*1 alleles (approximately 6%) were out of this range.

Most of the *CYP3A4* variants found in the coding region have an allele frequency <1%. An exception was CYP3A4*2, a SNP in exon 7 (15713T>C) that results in a Ser222Pro change with a frequency of 5% among the Caucasians but its effect on TAC bioavailability has not been established. This allele was linked to a lower clearance of the CYP3A4 substrate nifedipine, and carriers of this allele can thus be classified as “slow metabolizers” [34,35].

*ABCB1* (or *MDR-1*) is the gene codifying for P-glycoprotein, a transmembrane efflux pump moving drugs from the intracellular to the extracellular domain thereby influencing the absorption, cellular metabolism, and toxicity of pharmacological agents [36]. The most common and extensively studied *ABCB1* SNPs include a C to T transition at position 3435 within exon 26 (rs1045642), a C to T transition at position 1236 within exon 12 (rs1128503) and a G to T or A transition at position 2677 within exon 21 (rs2032582) of the *ABCB1* gene [37].

Influence of ABCB1 SNPs on TAC pharmacokinetics remains uncertain because several studies obtained conflicting results [29,38,39,40,41,42,43].

CsA is mainly metabolized by the CYP3A4 enzyme but neither polymorphisms in this gene, nor in the *ABCB1* gene, seem to have a strong effect on the dose to blood concentration ratio of CsA [44,45,46].

However, measurement of ABCB1 activity in PBMC of renal transplant recipients revealed that TT carrier patients on C3435T, G2677T, and C1236T SNPs showed lower ABCB1 activity than non-carriers [47]. A lower ABCB1 activity, particularly due to the 3435T variant allele causes an increased intracellular concentration of CsA thereby exposing the patients to a higher risk of drug-related adverse effects [48]. Other authors reported an association between reduction of intracellular CsA T-lymphocyte concentration and rejection episodes [49].

Interestingly, several studies have reported an association between *ABCB1* polymorphisms in donors and long-term graft survival. In particular, the TT variant allele at the 3435 position in *ABCB1* either in the donor or in the recipient were associated with decreased renal allograft function or graft loss in the long-term post-transplant [50,51,52,53,54,55,56]. In addition it has been reported that the *ABCB1* 1199G>A polymorphism is associated with better long-term renal function [57].

However, although most of the tacrolimus-related pharmacogentic studies are encouraging, the only published randomized controlled-study using this approach (TACTIC study) reported that adaptation of TAC dose according to the CYP3A5 genotype in renal transplant recipients did not reduce the incidence of delayed graft function, the number of post-transplant dialysis sessions per patient or the number of acute rejection episodes compared to the standard dose regimen [58].

### 2.2. Mycophenolate Mofetil (MMF)

MMF is a prodrug that is rapidly hydrolyzed to the active metabolite, mycophenolic acid (MPA), and acts by blocking nucleic acid synthesis through potent, selective, noncompetitive inhibition of inosine monophosphate dehydrogenase (IMPDH) a key enzyme of the *de novo* synthesis of guanosine nucleotides.

By inhibiting the *de novo* pathway for purine synthesis, a biochemical machinery utilized by lymphocytes, this agent is able to dramatically and selectively reduce B and T lymphocyte proliferation. In fact, other rapidly dividing cells are capable of recycling purine nucleotides through the “salvage” pathway, which is not inhibited by MPA [59].

MPA is metabolized to inactive 7-*O*-mycophenolic acid MPA glucuronide (MPAG) via hepatic UDP-glucuronosyltransferase (UGT), particularly UGT1A9, and is then deglucuronidated in the gastrointestinal tract and reconverted into MPA in the gut lumen where it is then reabsorbed through enterohepatic recirculation [59,60,61]. The acyl glucuronide (AcMPAG), is another MPA metabolite with immunosuppressive activity, formed by UGT2B7 [61]. MPA is extensively bound (97%–98%) to serum albumin. Accumulation of MPAG in patients with severe renal dysfunction has been shown to compete with free MPA for binding to albumin, leading to an increase in the serum concentration of free MPA [44,62].

There are currently no definitive pharmacogenetic associations that have been identified for MPA, although some potential associations between specific polymorphisms and pharmacokinetic and incidence of adverse effects appear promising (Table 1).

In particular, several reports have shown that two SNPs in the promoter region of the *UGT1A9* gene, −2152C>T and −275T>A are associated with an enhanced glucuronidation of MPA to MPAG [63] and significantly reduced MPA levels in the early phase after renal transplantation [64,65].

Van Schaik *et al.* [66] confirmed that the *UGT1A9* −275T>A and/or −2152C>T polymorphisms were associated with an average 20% lower MPA exposure (consistent with an higher enzymatic activity) and with a significantly increased risk of acute rejection in fixed-dose MMF-treated patients who received TAC; the effect of these polymorphisms on MPA level were confirmed by other researchers [63,64,67,68].

The less frequent UGT1A9*3 SNP, present in less than 5% of the white population, was associated with a higher MPA exposure, in accordance with a reduction of *in vitro* enzymatic activity [63,64,65].

Although polymorphisms in other genes have also been reported to be associated with MPA pharmacokinetics, it is well accepted that SNPs in *UGT1A9* have the strongest influence [64,69,70].

Furthermore, polymorphisms in genes associated with drug transport (including *ABCB1* and *SLCO1B1*) or drug metabolism (*UGT1A8* and *UGT2B7*) have been linked to adverse events, especially diarrhoea and haematological adverse effects, in patients treated with MMF [71,72,73,74,75,76]. However, none of these studies have been translated into clinical practice.

Additionally a part of the pharmacogenetics of MMF has been focused on IMPDH, a rate-limiting enzyme in the *de novo* pathway of guanosine nucleotide synthesis. IMPDH exists in two isoforms IMPDH-1 and IMPDH-2 derived from different genes and several SNPs have been reported [77,78,79,80].

An association has been shown between high IMPDH activity prior to transplant and acute transplant rejection [81], suggesting that patients with high activity may theoretically need more MMF for equivalent immunosuppression. Patients with low IMPDH activity require a lower MMF dosage to obtain the same immunosuppressive effect [81].

Wang *et al.* [80,82] reported that the rs2278293 and rs2278294 SNPs within intron 7 of *IMPDH-1* are significantly associated with the incidence of biopsy-proven acute rejection one year after renal transplantation. Although the mechanism of this association has not been determined, the authors suggest the possible linkage to other SNPs [80].

*IMPDH-2* is more conserved than *IMPDH-1* and an association was found between 3757T>C polymorphism and an increased enzymatic activity in MMF-treated renal transplant patients [83].

Patients with at least one variant *IMPDH-2* 3757C allele had a mean IMPDH activity 48% higher compared with *IMPDH-2* 3757TT wild-type patients although there is conflicting data evaluating the impact of this variant on acute rejection risk [84].

Shah *et al.* [85] in 2012, analyzing 1000 renal transplant recipients, found no association between IMPDH variants and renal allograft rejection and graft survival.

Overall, there is currently not enough data to suggest that routine pharmacogenomic testing would enhance patient outcomes in relation to mycophenolate safety and efficacy.

### 2.3. Mammalian Target of Rapamycin Inhibitors (mTOR-Is)

The mTOR-Is, sirolimus and everolimus, represent a class of proliferation signal inhibitors used in renal transplantation with a wide spectrum of activities, including suppression of T-cell proliferation and reduction of tumor growth.

The main mechanism of action of these drugs is the inhibition of the mammalian target of rapamycin (mTOR). mTOR is a regulatory protein kinase involved in lymphocyte proliferation, developmental processes such as neurologic and muscle generation, and tumor cell growth. Sirolimus (SRL; Rapamune, Wyeth Pharmaceuticals, New York, NY, USA) was the first mTOR-I approved for use in renal transplant recipients and binds to FKBP-12. Everolimus (EVR), marketed as Certican, was recently approved and is structurally similar to SLR but with the addition of an extra hydroxyethyl group at position 40. Although the pharmacokinetic characteristics are similar between the two mTOR inhibitors, EVR was developed in an attempt to improve the pharmacokinetic characteristics of SRL and oral bioavailability with a shorter elimination half-life that necessitates twice-daily dosing *versus* once-daily dosing [86].

EVR and SRL are metabolized primarily by the CYP3A family of enzymes [87,88] and ABCB1.

Several studies have investigated the influence of SNPs in *CYP3A4* and *CYP3A5* genes on the dose requirement and clearance of the immunosuppressant (Table 1).

In particular, lower SRL concentration/dose ratio was observed in the CYP3A5*1 (*CYP3A5* expresser) carriers than in the CYP3A5*3/*3 carriers (non-expressers), suggesting that *CYP3A5* non-expressers require a lower SRL daily dose to achieve adequate blood concentration. Patients with CYP3A5*1/*1 are more likely to have a higher liver metabolism and require a higher daily dose to achieve adequate blood SRL levels [89,90].

Moreover, patients carrying the CYP3A4*1B (−392A>G) allele, being associated with a higher enzymatic activity, require higher SRL doses to achieve adequate blood concentrations [89].

These results were found only for SRL, but not in all studies, and only in patients not treated with CNIs [91,92,93].

Recently, new polymorphisms have been discovered: CYP3A4*22, in intron 6 of *CYP3A4* gene, POR*28 (P450 (cytochrome) oxidoreductase) (rs1057868-C>T), and *PPARA* (peroxisome proliferator-activated receptor alpha) (rs4253728-G>A), but they do not have any impact on the pharmacokinetics of SRL or the occurrence of SRL adverse events in kidney transplant recipients [94].

Moreover, Sam *et al.* [95] reported that the mean SRL concentration:dose ratio was 48% higher in patients with the *ABCB1* 3435CT/TT genotype than in those with the 3435CC genotype, and was 24% higher in *IL-10* −1082GG homozygotes than in those with −1082AG/AA, consistent with enhanced IL-10 expression leading to lowered CYP3A activity and reduced SRL metabolism in patients with this genotype [96,97]. In another study the same authors suggest an association between the same SNPs and higher triglyceride levels after SRL treatment [98].

Similarly to CNIs, mTOR inhibitors carry several adverse effects (including hypertriglyceridemia, hyperlipidemia, edema, pulmonary fibrosis, anemia) and they may potentiate nephrotoxicity with increased urinary protein excretion [99,100].

### 2.4. Azathioprine (AZA)

AZA is one of the older immunosuppressive medications used in nephrology and in the treatment of inflammatory bowel disease and several autoimmune diseases. It is converted mainly in the liver into 6-mercaptopurine (6-MP), possibly as a result of a glutathione-*S*-transferase (GST) catalyzed reaction [101]. Conversion of 6-MP by the enzyme hypoxanthine guanine phosphoribosyltransferase leads to the formation of 6-thioguanine-nucleotides (6-TGN), that, when incorporated into DNA, are directly cytotoxic and also suppress *de novo* purine synthesis [102]. The thiopurine methyl-transferase (TPMT) pathway leads to the methylation of 6-MP forming 6-methylmercapotpurine, an inactive form of AZA [103,104]. Thus, the metabolism of thiopurines by TPMT shunts the drug down the methylation pathway and away from the active pathway [105].

This enzyme is encoded by the *TPMT* gene and its activity exhibits genetic polymorphism: approximately 90% of individuals inherit high activity, 10% have intermediate activity because of heterozygosity, and 0.3% have low or no detectable enzyme activity because they inherit two nonfunctional TPMT alleles [106].

To date, 20 variant alleles (TPMT*2-*18) have been identified, which are associated with decreased activity compared with the TPMT*1 wild-type allele [107]. Three alleles—TPMT*2, *3A, and *3C—account for up to 95% of intermediate or low enzyme activity cases [108]. The pattern and frequency of mutant *TPMT* alleles is different among various ethnic populations [109]. The TPMT*3A allele contains two point mutations: G460A in exon 7 and A719G in exon 10 that lead to Ala154Thr and Tyr240Cys amino acid substitutions, TPMT*3C has only a single A719G transversion, and TPMT*2 contains a G238C transversion, producing Ala80Pro substitution [110,111]. Other identified variant alleles are very rare (*3B) or were found only in single individuals (*4–*18) [112].

The impaired or absent ability to metabolize AZA through TPMT leads to high blood levels of TGN and an increased risk of developing severe and potentially life-threatening myelotoxicity when no dose reductions are performed [113,114,115,116,117,118] (Table 1). Dervieux *et al.* [119], measuring the TPMT activity in red blood cells of pediatric patients after renal transplantation, demonstrated that elevated TPMT activity was associated with an increased risk of acute rejection.

Moreover Thervet *et al.* observed that TPMT induction (caused by the rise of the concentration of its substrate, 6-MP) is associated with a lower incidence of clinical acute rejection [120].

Genotyping for *TPMT* polymorphisms, before initiation of AZA therapy is one of the few examples of a pharmacogenetic test that has made the transition from research into clinical practice, especially for autoimmune disease [121]. This testing improves safety by avoiding full-dose treatment in patients with a (partial) enzyme deficiency [118].

Owing to the association between homozygous and heterozygous genotypes and low to moderate TMPT activity leading to increased bone marrow suppression, the Clinical Pharmacogenetics Implementation Consortium Guidelines for Thiopurine Methyltransferase Genotype and Thiopurine Dosing endorse genotyping prior to initiating AZA therapy to inform dosing or for selection of alternate therapy [122].

**Table 1:** Gene polymorphisms and their effects.

Drug	Gene	Polymorphism	Biological Effect	Clinical Effect	References
Tacrolimus (TAC)	*CYP3A5*	CYP3A5*3 (6986A>G)	Reduction of CYP3A5 activity	Reduced TAC dose requirement	[24,25,26,27]
	*CYP3A4*	CYP3A4*22	Reduction of CYP3A4 activity	Reduced TAC dose requirement	[31,32]
		CYP3A4*1B (392A>G)	Increment of CYP3A4 activity	Increased TAC dose requirement	[33]
	*ABCB1*	3435C>T	Altered ABCB1 activity	Influence on TAC dose requirement is uncertain	[29,38,39,40,41,42,43]
		1236C>T			[38,39,40,41,42,43]
		2677G>T/A			[38,39,40,41,42,43]
Ciclosporin (CsA)	*ABCB1*	3435C>T	Reduction of ABCB1 activity	Increased CsA intracellular concentration; TT variant is associated with CsA nephrotoxicity and long-term graft survival	[48,50,51,52,53,54,55,56]
Mycophenolate mofetil/Mycophenolic acid (MMF/MPA)	*UGT1A9*	2152C>T275T>A	Increment of UGT1A9 activity	Increased risk of acute rejection	[66]
		UGT1A9*3	Reduction of UGT1A9 activity	Influence on MPA pharmacokinetics	[64,65]
	*IMPDH-1*	rs2278293	Most likely associated with an increment of IMPDH activity	Probably associated with the incidence of biopsy-proven acute rejection	[80,82]
		rs2278294			
	*IMPDH-2*	3757T>C	Increment of IMPDH activity	No association with rejection risk	[84]
Sirolimus (SRL)	*CYP3A5*	CYP3A5*3	Reduction of CYP3A5 activity	Reduced SRL dose requirement	[89,90]
	*CYP3A4*	CYP3A4*1B (392A>G)	Increment of CYP3A4 activity	Increased SRL dose requirement	[89]
	*ABCB1*	3435C>T	Reduction of ABCB1 activity	Patients 3435CT/TT have increased SRL concenttration:dose ratio	[95]
Everolimus (EVR)	*CYP3A5*	CYP3A5*3	Reduction of CYP3A5 activity	No impact on EVR pharmacokinetics	[92]
Azathioprine (AZA)	*TPMT*	TPMT*2	Reduction of TPMT activity	High risk of myelotoxicity	[113,114,115,116,117,118]
		TPMT*3A			
		TPMT*3B			
		TPMT*3C			

## 3. Pharmacogenomics: Looking to the Polygenetic Influence in the Response to Drug Therapy

The completion of the Human Genome Project [123,124] and the development of innovative high-throughput screening technologies (e.g., genome wide scans, haplotype analysis and candidate gene approaches) has led to the development of pharmacogenomics, a new science that, screening the entire genome, is able to recognize determinants of drug responses or toxicities.

The Food and Drug Administration (FDA) has defined pharmacogenomics as “the study of variations of DNA and RNA characteristics as related to drug response”, whereas “pharmacogenetics” is “the study of variations in DNA *sequence* as related to drug response” [125]. More specifically, pharmacogenomics evaluates molecular determinants at the genome-, transcriptome-, and proteome-wide levels, whereas pharmacogenetics involves limited and specific genetic markers [126,127].

The use of pharmacogenetics/pharmacogenomics in clinical practice to personalize the dosage of a specific medication avoiding/minimizing its toxicity and enhancing its therapeutic effects, is referred as “personalized medicine”.

Pharmacogenomic data may be integrated in the labeling of a product determining a clear identification of patients who require dose adjustments for the prevention of adverse effects or lack of efficacy. The FDA has approved inclusion of pharmacogenetic data in the labelling of drugs like warfarin [128] and irinotecan [129] mainly for safety concerns.

Pharmacogenomic data could also be utilized by the pharmaceutical industries as a part of drug development [130].

However a genetic or genome profile alone is not the only factor influencing a drug response for a disease: other factors such as age, gender, body mass, potential drug-drug interaction need to be considered.

PharmGKB [131] is the leading pharmacogenetics and pharmacogenomics knowledge base (available online: http://www.pharmgkb.org) for annotation, integration, and aggregation of pharmacogenetic/pharmacogenomic knowledge through relationships with other resources, such as the University of California Santa Cruz (CA, USA) Genome Browser [132], Drugbank [133] and Biopax [134].

To obtain the above-indicated results, pharmacogenomic research strategies need to be accurately and rigorously conducted. Several commercial techniques are currently available and researchers may choose the most appropriate platform to use in their projects. Among them, the DNA microarray (also referred to as gene or genome chip, DNA chip or biochip) represents the most utilized technique.

Although there have been numerous clinical studies and molecular genetics research in this area, we are still far from the possibility of extensive use of pharmacogenomics in clinical practice, primarily because the clear-cut demonstration that genotype-based dosing could definitely improve clinical outcomes is still lacking. In addition it is noteworthy that several barriers exist: economic, educational and legal. The evidence that the cost of pharmacogenomics test could be justified by clinical outcome is lacking. Medical students and practicing physicians must be educated and trained to use pharmacogenetic tests and properly interpret their clinical relevance [135,136].

Moreover, the need for complex data analysis and bioinformatics represents an additional obstacle to the expansion of “genomic medicine” [137]. Therefore, further studies are necessary to obtain more simple algorithms and statistical methods to discover genes variants or target genes influencing drug therapies.

However, at the moment, only few “omics” research approaches have been undertaken in renal transplant medicine and, mostly, they have not been translated in “day to day” clinical practice.

Among them, the Dutch Pharmacogenetics Working Group and Pharmacogenomics Knowledge Base have recently published dose recommendations based on TPMT genotype [138]. Additionally, as previously mentioned, the Clinical Pharmacogenetics Implementation Consortium has published guidelines on the use of TPMT genotyping in clinical practice [122].

Interstingly, also, the DeKAF study, a pharmacogenomic analysis of 945 Kidney transplant recipients has shown that a number of SNPs in several genes were associated with early CsA-related nephrotoxicity [139].

## 4. Research on New Therapeutic Targets for Immunosuppression

In the last decade, nephrology researchers have started to employ “omics” techniques to select new potential therapeutic targets for immunosuppression.

In 2003 Sarwal *et al*. [140] demonstrated that a systematic analysis of gene-expression patterns was able to reveal a specific biology and pathogenesis fingerprints of renal allograft rejection. Biopsy samples from patients with acute rejection that are indistinguishable on conventional histologic analysis demonstrated extensive differences in gene expression, which are associated with differences in immunologic and cellular features and clinical course. The presence of dense clusters of B cells in a biopsy sample was strongly associated with severe graft rejection, suggesting a pivotal role of infiltrating B cells in acute rejection. Based on these results, authors speculated that in patients who have such an infiltration, early treatment with a monoclonal antibody against CD20 (rituximab) could be beneficial.

Subsequently, a similar methodological approach has been undertaken to recognize biological elements specifically involved in the development of immunological tolerance in renal transplantation. Some transplant recipients, in fact, display a stable graft function without immunosuppression namely “operational tolerance” [141,142]. The identification of “biomarkers of tolerance” could enable physicians to recognize patients in which immune therapy could be minimized or interrupted. The “tolerogenic” signature currently suggested, contains genes encoding for protein implicated in apoptosis, immune quiescence and T cell responses [141].

More recently Newell *et al*. [143], through microarray technology and functional analysis found that tolerant patients exhibited increased numbers of total and naive B cells and had enhanced expression of B cell differentiation and activation genes compared with subjects receiving immunosuppression. In particular they found three genes (*IGKV4-1*, *IGLLA*, and *IGKV1D-13*) that predicted tolerance with 100% accuracy. These genes are all expressed during the differentiation of B cells from pre- to mature B cells or during B cell activation-induced transition. They suggest that transitioning or maturing B cells are involved in tolerance induction and/or maintenance.

However, additional studies are required to assess the clinical utility of the above-identified biological/cellular factors.

Recently, our group, utilizing a high-throughput research strategy combined with classical bimolecular methodologies, revealed that neutral endopeptidase (NEP), an enzyme that catalyzes the degradation of a number of endogenous vasodilator peptides, such as angiotensin-II, was significantly up-regulated in renal transplant recipients after conversion from AZA to MPA. Immunohistochemical analysis confirmed results obtained by microarray and revealed that glomerular NEP was inversely correlated with glomerulosclerosis and proteinuria, while tubular NEP was inversely associated with interstitial fibrosis. These results suggest that an MPA-induced up-regulation of NEP could decrease proteinuria and delay the progression of chronic renal damage in renal transplant recipients [144].

Furthermore, a combined strategy between classical biomolecular strategies and high-throughput technologies was able to identify, for the first time, that karyopherins, adaptor proteins that recognize the first discovered or classical NLS, may have a pivotal role in the development of delay graft function (DGF), and that these molecules may be new valuable therapeutic targets. In fact, in the last years, several drugs have been studied as inhibitors of karyopherin trafficking (e.g., importazole, Ivermectin) [145,146,147,148].

Also, several studies employing microarray methodologies have been undertaken to select potential pharmacological targets useful to minimize the onset of acute rejection.

Kainz *et al.* [149], performing a complex genome-wide analysis using nucleic materials isolated from graft tissues obtained before and one-year post-transplantation, identified 52 genes able to accurately discriminate patients with excellent *versus* poor renal function. Up-regulated genes in patients with reduced graft function were involved in immunity and defense, signal transduction and response to oxidative stress, while down-regulated genes were mainly involved in metabolism, ion binding and transport.

Finally, in a recent paper Saint-Mezard *et al.* [150], through a comparative analysis among three different microarray datasets (GSE343, GSE9493 and GSE1563), identified a specific and early diagnostic “acute rejection transcript set” (including 70 genes).

## 5. Conclusions

The correct administration of immunosuppressive therapy has a significant impact in graft and patients’ survival. Several *in vivo* and *in vitro* studies underline that dosage has an important role in minimizing chronic graft damage, but unfortunately, except for the evaluation of trough drug levels, no clinically useful tools are currently available to correctly manage immunosuppressive therapy.

**Figure 3:**
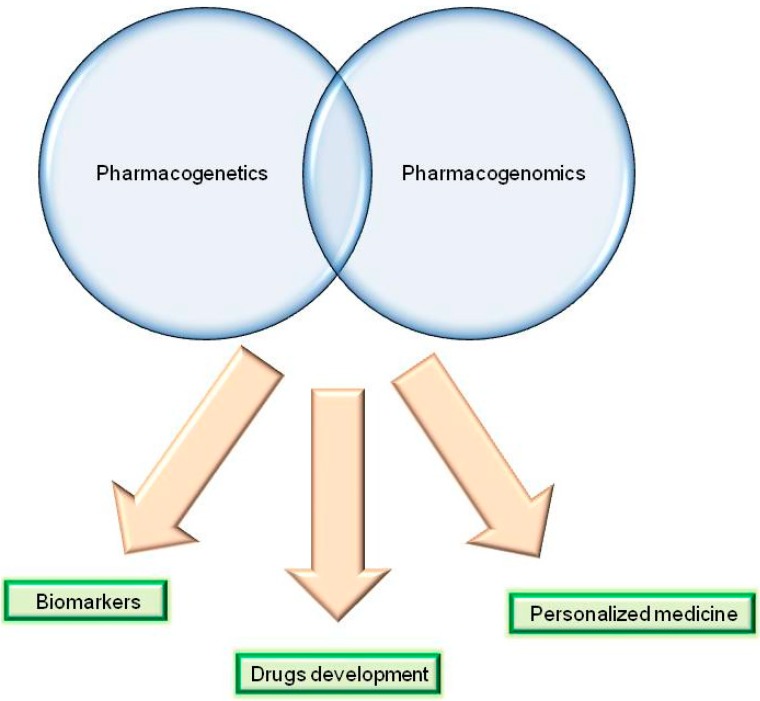
Prospective employment of pharmacogenetics and pharmacogenomics research strategies.

For this purpose, we believe that “omics” techniques represent powerful methods that could be routinely employed in the future to aid clinicians in the personalized treatment of patients to avoid severe toxicities/adverse effects and increase therapeutic potential (Figure 3).

We are, however, still far from a realistic clinical employment of these strategies in nephrology and organ transplant medicine, and it seems reasonable that different professionals (physicians, scientists) should work together to rapidly overcome the scientific, economic, educational and legal barriers [151] that still exist before personalized medicine is achieved.
